# Dynamic miRNA Landscape Links Mammary Gland Development to the Regulation of Milk Protein Expression in Mice

**DOI:** 10.3390/ani12060727

**Published:** 2022-03-14

**Authors:** Wenjing Wang, Xupeng Zang, Yonglun Liu, Yunyi Liang, Gengyuan Cai, Zhenfang Wu, Zicong Li

**Affiliations:** 1 National Engineering Research Center for Breeding Swine Industry, South China Agricultural University, Guangzhou 510642, China; wwenjmail@163.com (W.W.); xupeng_zang@163.com (X.Z.); lylyonglun@163.com (Y.L.); yunyi13321@163.com (Y.L.); cgy0415@163.com (G.C.); 2 Department of Animal Genetics, Breeding and Reproduction, College of Animal Science, South China Agricultural University, Guangzhou 510642, China; 3 Guangdong Provincial Key Laboratory of Agro-Animal Genomics and Molecular Breeding, South China Agricultural University, Guangzhou 510642, China; 4 State Key Laboratory for Conservation and Utilization of Subtropical Agro-Bioresources, South China Agricultural University, Guangzhou 510642, China; 5 Guangdong Provincial Laboratory of Lingnan Modern Agricultural Science and Technology, Guangzhou 510642, China

**Keywords:** mammary gland, mouse, microRNA, milk protein

## Abstract

**Simple Summary:**

Milk synthesis is vital for maintaining the normal growth of newborn animals. Abnormal mammary gland development leads to a decrease in female productivity and the overall productivity of animal husbandry. This study characterized the dynamic miRNA expression profile during the process of mammary gland development, and identified a novel miRNA regulating expression of β-casein—an important milk protein. The results are valuable for studying mammary gland development, increasing milk production, improving the survival rate of pups, and promoting the development of animal husbandry.

**Abstract:**

Mammary gland morphology varies considerably between pregnancy and lactation status, e.g., virgin to pregnant and lactation to weaning. Throughout these critical developmental phases, the mammary glands undergo remodeling to accommodate changes in milk production capacity, which is positively correlated with milk protein expression. The purpose of this study was to investigate the microRNA (miRNA) expression profiles in female ICR mice’s mammary glands at the virgin stage (V), day 16 of pregnancy (P16d), day 12 of lactation (L12d), day 1 of forced weaning (FW1d), and day 3 of forced weaning (FW3d), and to identify the miRNAs regulating milk protein gene expression. During the five stages of testing, 852 known miRNAs and 179 novel miRNAs were identified in the mammary glands. Based on their expression patterns, the identified miRNAs were grouped into 12 clusters. The expression pattern of cluster 1 miRNAs was opposite to that of milk protein genes in mammary glands in all five different stages. Gene Ontology (GO) and Kyoto Encyclopedia of Genes and Genomes (KEGG) analyses revealed that the predicted target genes of cluster 1 miRNAs were related to murine mammary gland development and lactation. Furthermore, fluorescence in situ hybridization (FISH) analysis revealed that the novel-mmu-miR424-5p, which belongs to the cluster 1 miRNAs, was expressed in murine mammary epithelial cells. The dual-luciferase reporter assay revealed that an important milk protein gene—β-casein (*CSN2*)—was regarded as one of the likely targets for the novel-mmu-miR424-5p. This study analyzed the expression patterns of miRNAs in murine mammary glands throughout five critical developmental stages, and discovered a novel miRNA involved in regulating the expression of *CSN2*. These findings contribute to an enhanced understanding of the developmental biology of mammary glands, providing guidelines for increasing lactation efficiency and milk quality.

## 1. Introduction

The mammary gland is an epidermal appendage that has evolved over 300 million years, and distinguishes mammals from all other animals due to its distinctive anatomical structure [1]. The mammary gland is a complex and unique glandular organ that only develops fully after birth [2]. When females reach puberty, the rudimentary ductal system that has existed since the embryonic period starts to formally develop and fill the fat pad [3]. Subsequently, during pregnancy, the gland undergoes numerous changes, including ductal proliferation and lobuloalveolar differentiation under the combined action of progesterone and prolactin. During lactation, alveoli generated via mammary differentiation secrete milk. The lack of demand for milk at weaning triggers the process of involution, whereby the gland remodels the epithelial tree back to a simple ductal architecture [4]. The mammary gland undergoes obvious remodeling throughout these stages, including the biological processes of cell proliferation, differentiation, and apoptosis. Several hormones, molecular signaling pathways, and transcription factors have been found to control these processes [5,6,7].

The function of the mammary gland is to produce and secrete copious quantities of milk to provide sufficient nutrients for maintaining the survival and normal development of newborns. Anomalies in the development of the mammary gland result in decreased milk production, thus limiting the growth and survival of offspring [8]. Milk protein is a significant component of milk [9]. Previous studies have shown that β-casein (*CSN2*), αs1-casein (*CSN1S1*), and whey acidic protein (*WAP*) are the major proteins in milk, and are believed to be markers for mammary secretory differentiation [10,11]. Milk protein genes are responsible for controlling milk production [12]. Although earlier studies have shown that multiple factors control the expression of milk protein genes [13,14], the molecular mechanism regulating milk protein gene expression is still not fully known.

MicroRNAs (miRNAs) belong to a class of small non-coding RNAs (20~24 nucleotides), which are among the most abundant types of gene expression regulators in mammals [15]. Numerous studies have shown the importance of miRNAs in mammary gland biology to date. MiR-223, for example, has been shown to regulate the growth and morphogenesis of luminal mammary epithelial cells (MECs) [16], while miR-15b regulates lipid synthesis and milk production in MECs [17]. Despite considerable research exploring the role of miRNAs in mammary gland development, the expression profiles and functions of miRNAs throughout mammary development, along with the regulation of the expression of milk protein genes, remain largely unknown.

The purpose of this study was to investigate the expression patterns of miRNAs during murine mammary gland development, and to identify miRNAs that control the expression of milk protein genes. Mammary gland tissues were obtained from female ICR mice at five distinct stages, including mature virgin (V), day 16 of pregnancy (P16d), day 12 of lactation (L12d), day 1 of forced weaning (FW1d), and day 3 of forced weaning (FW3d). miRNAs that link mammary gland developmental stages to the regulation of milk protein gene expression were identified using small RNA sequencing.

## 2. Materials and Methods

### 2.1. Animals and Mammary Gland Tissues Collection

This study was approved by the Ethics Committee of the Laboratory Animal Center of South China Agricultural University (permit number: SYXK-2019-0136). Fifteen specific-pathogen-free (SPF) female ICR mice and eight male ICR mice, all aged approximately 7 weeks and of similar weight, were provided by Liaoning Changsheng Biotechnology Co., Ltd. (Benxi, Liaoning, China). These mice were housed under a 12 h light/dark cycle in a 22 °C environment, with free access to food and water.

Fifteen female mice were randomly allocated into five groups (n = 3 per group): mature virgin (V) at the age of 8-10 weeks, day 16 of pregnancy (P16d), day 12 of lactation (L12d), day 1 of forced weaning (FW1d), and day 3 of forced weaning (FW3d). Female mice, except for those in the V stage, were paired with males for mating, and were examined for the presence of a vaginal plug the morning following pairing. When a vaginal plug was observed, the date was considered to be day 0 of pregnancy (P0d), and the day of delivery was regarded as day 1 of lactation (L1d). Lactating mice were forced to be weaned at L12d, and the day of removing the suckling pups was regarded as day 0 of forced weaning (FW0d). The first and third days of weaning were regarded as day 1 of forced weaning (FW1d) and day 3 of forced weaning (FW3d). All female mice were killed by cervical dislocation under i.p. sodium pentobarbital anesthesia (45 mg/kg of BW) in order to obtain mammary gland tissue samples. Following the removal of lymph nodes, the abdominal and inguinal mammary glands were collected, rapidly frozen using liquid nitrogen, and stored at −80 °C [18]. Meanwhile, a portion of the inguinal mammary gland was fixed in 4% paraformaldehyde solution for 12 h for subsequent experiments.

### 2.2. Hematoxylin and Eosin Staining (H&E)

The conventional H&E staining technique was used [19]. Briefly, the fixed tissues were embedded, sectioned, deparaffinized, and rehydrated before being stained with hematoxylin and eosin solutions, decolorized in alcohol, and transparentized in xylene. Finally, the tissue sections were fixed with neutral gum so that the cell morphology could be examined using a Nikon 80i microscope (Nikon, Tokyo, Japan).

### 2.3. Whole-Mount Analysis

Following a previously reported whole-mount analysis method [20], the inguinal mammary glands were excised, spread on glass slides, and fixed overnight at room temperature in Carnoy’s fixative (i.e., 100% ethanol, chloroform, and glacial acetic acid at a 6:3:1 ratio). After removing the mammary glands from the fixative, they were immersed in 70% ethanol for 15–30 min. Subsequently, the glands were gradually rehydrated in water by removing 1/3 of the ethanol and replacing it with water. This step of adding water was repeated 3 times, with the glands soaking for ~5 min each time. The glands were soaked in water for 5 min before being stained in carmine alum solution (STEMCELL Technologies, Vancouver, BC, Canada) for 12–24 h, washed in 70%, 95%, and 100% ethanol for 15 min each, cleared in xylene, and mounted. A Nikon SMZ1000 stereoscopic microscope (Nikon, Tokyo, Japan) was used to examine the mammary gland tissues.

### 2.4. Total RNA Extraction and Small RNA Library Construction

Total RNA was extracted from 15 mammary gland tissue samples using TRIzol reagent (Invitrogen, Carlsbad, CA, USA), according to the manufacturer’s instructions. Subsequently, RNA purity was determined using a NanoDrop ND2000 spectrophotometer (Thermo Fisher Scientific, Waltham, MA, USA) at 260 and 280 nm, and the RNA integrity number (RIN) was assessed with an Agilent 2100 Bioanalyzer (Agilent Technologies, Santa Clara, CA, USA). To construct the library, ~3 μg of total RNA was extracted from each sample. Total RNA was separated on a 15% urea denaturing polyacrylamide gel electrophoresis (PAGE) gel, and small RNA regions corresponding to the 18–30 nt bands in the labeled lane (14–30 ssRNA Ladder Marker, TAKARA, Dalian, China) were excised and recovered. Subsequently, the isolated small RNAs were linked with adenylated 3′ adapters annealed to unique molecular identifiers (UMI), followed by 5′ adapter ligation. The adapter-ligated small RNAs were then reverse-transcribed into cDNA using SuperScript II Reverse Transcriptase (Invitrogen, Carlsbad, CA, USA), and the cDNA fragments were enriched using reverse-transcription polymerase chain reaction (forward primer: 5′-GAACGACATGGCTACGA-3′ and reverse primer: 5′-TGTGAGCCAAGGAGTTG-3′). In the reverse-transcription system, the reaction conditions were 42 °C for 1 h, and 70 °C for 15 min. In addition, the reaction conditions of the PCR amplification system were 95 °C for 3 min, and then 15–18 cycles of (98 °C for 20 s, 56 °C for 15 s, 72 °C for 15 s) and, finally, 72 °C for 10 min, and held at 4 °C. The cDNA fragments were selected for target fragments of 110~130 bp using agarose gel electrophoresis, and then purified using the QIAquick Gel Extraction Kit (QIAGEN, Valencia, CA, USA). The DNBseq platform (BGI-Shenzhen, China) was used to sequence the final cDNA; 50 bp single-end reads were generated for further analyses.

### 2.5. Processing of Sequencing Data

After the high-throughput small RNA sequencing was completed, the raw tags (raw reads) were treated as follows: low-quality tags (where the number of bases with a Phred value less than 10 is less than or equal to 4, and the number of bases with a Phred value less than 13 is less than or equal to 6) were removed; tags with 5′ adapter contaminants were removed; tags without a 3′ adapter were removed; tags without an insert were removed; tags with poly A were removed; tags shorter than 18 nt were removed. Following filtering, the clean tags were mapped to the reference genome using Bowtie2 (http://bowtie-bio.sourceforge.net/index.shtml, accessed on 15 February 2021, with -q -L 16 --phred64 -p 6) [21] to predict miRNAs and small other non-coding RNAs. Furthermore, reads were compared with non-coding RNA and Rfam databases, and non-coding RNA annotations were performed on sequencing data. For Rfam mapping, cmsearch (https://omictools.com/cmsearch-tool, accessed on 20 February 2021, with --cpu 6 --noali) [22] was used. The software miRDeep2 (https://github.com/rajewsky-lab/mirdeep2, accessed on 20 February 2021, with default parameters) [23] was used to predict novel miRNAs by examining the distinctive secondary structures.

### 2.6. Clustering of miRNA Profiles

The Mfuzz package (v.2.54.0) implemented in R was used to perform cluster analysis of miRNAs using default parameters [24]. The miRNAs with consistent expression patterns were clustered, and their expression profiles were revealed using the c-means method and drawing line graphs of the expression levels of miRNAs detected in all samples at the five developmental stages.

### 2.7. Prediction and Functional Annotation Analysis of Target Genes of miRNAs

TargetScan (v.7.0, Cambridge, MA, USA, with default parameters) [25], miRanda (v.3.3, New York, NY, USA, with -sc 150 -en -20) [26], and RNAhybrid (v.2.1.2, Bielefeld, Germany, with -m 100,000 -*p* 0.05) [27] were used to identify miRNAs’ target genes, and the overlapping results predicted by the three programs were considered to be the predicted miRNAs’ target genes. The miRNA–gene regulatory networks were structured using Cytoscape software (v.3.7.2, La Jolla, CA, USA, http://www.cytoscape.org/, accessed on 15 April 2021) [28] to reveal the interactions between miRNAs and their target genes. Gene Ontology (GO) enrichment and Kyoto Encyclopedia of Genes and Genomes (KEGG) pathway analyses were performed on miRNAs’ target genes to better understand their potential biological functions and primary pathways. GO terms and KEGG pathways of miRNAs and the target genes of the miRNAs from individual clusters were enriched using the clusterProfiler R package (v.3.10.1) [29]

### 2.8. Quantitative Real-Time PCR (qPCR)

The Evo M-MLV RT Kit with gDNA Clean for qPCR II (Accurate Biology, Changsha, China) was used to synthesize the first-strand cDNA. The qPCR was then performed in triplicate using the SYBR^®^ Green Premix Pro Taq HS qPCR Kit (Accurate Biology, China) in a real-time PCR system (Applied Biosystems, Foster City, CA, USA) to determine the expression of mRNAs, following the manufacturer’s protocol. Table S1 lists the primers for three milk protein genes, and the relative quantification of gene expression was normalized to that of the endogenous gene *β-actin*. To validate the authenticity of the miRNA-Seq results, 8 miRNAs were randomly selected for qPCR using the stem–loop qPCR technique [30]. Table S1 displays the primer sequences for the selected miRNAs. For the normalization studies, murine U6 snRNA was used as an internal control, and all reactions were run with three technical replicates [31]. The comparative cycle threshold (2^−∆∆Ct^) method was used to determine the relative expression levels of the miRNAs.

### 2.9. Fluorescence In Situ Hybridization (FISH) Analysis

FISH was performed on the mammary tissues at the five stages to determine the location of novel-mmu-miR424-5p, as described in a previously published study [32]. In brief, paraffin-embedded mammary tissue blocks were sectioned at 4-micrometer thickness, deparaffinized, digested with proteinase K, and then hybridized with a novel-mmu-miR424-5p FAM (green)-labeled probe. Meanwhile, DAPI was used to stain the nuclei. All procedures were performed according to the manufacturer’s instructions, and then fluorescent images were captured with the use of a positive fluorescence microscope (Nikon, Tokyo, Japan).

### 2.10. Dual-Luciferase Reporter Assay

The *CSN2* 3′UTR as wild-type (WT) sequences containing the putative novel-mmu-miR424-5p binding sites, along with mutant (Mut) sequences, were inserted downstream of the luciferase gene in the pmirGLO vector (Promega Corporation, Madison, WI, USA). The 293T cells were seeded into 24-well plates at 1 × 10^5^ cells/well for luciferase reporter assay. *CSN2* 3′UTR or *CSN2* 3′UTR-Mut, as well as novel-mmu-miR424-5p mimics and NC mimics (sense: 5′-UUCUCCGAACGUGUCACGUTT-3′ and antisense: 5′-ACGUGACACGUUCGGAGAATT-3′), were co-transfected into 293T cells using Lipofectamine 3000 (Thermo Fisher Scientific, Waltham, MA, USA), according to the manufacturer’s instructions. The firefly and *Renilla luciferase* activities were determined in co-transfected cells 48 h after co-transfection using the Dual-Luciferase Reporter Assay Kit (Beyotime Biotechnology, Shanghai, China). Finally, firefly-to-*Renilla luciferase* ratios for each well were computed, and each experiment was performed in triplicate.

### 2.11. Statistical Analysis

Graphs and statistical analysis of luciferase reporter assay data were created using GraphPad Prism 8.0 (GraphPad Software, San Diego, CA, USA). The data obtained were tested with the Kolmogorov–Smirnov test for normal distribution using SPSS 26.0, and comparisons between two groups were evaluated using Student’s *t*-test. All experiments were performed in three independent replicates, and all of the data were expressed as the mean ± the standard error of the mean (SEM); *p* < 0.05 was considered to be statistically significant, and *, **, and *** indicate *p* < 0.05, *p* < 0.01, and *p* < 0.001, respectively.

## 3. Results

### 3.1. Overview of the Small RNA Sequencing Data

We performed H&E staining and whole-mount analysis (Figure S1A) for collected mammary gland tissues at five different stages. Meanwhile, we investigated the expression patterns of three milk protein genes—*WAP*, *CSN2*, and *CSN1S1*—by qPCR at all five stages (Figure S1B). The findings were consistent with the previously reported histological changes in murine mammary development, confirming the accuracy of tissue samples obtained at each developmental stage. Small RNA libraries were generated from a total of 15 samples of V, P16d, L12d, FW1d, and FW3d murine mammary gland tissues.

**Figure 1:**
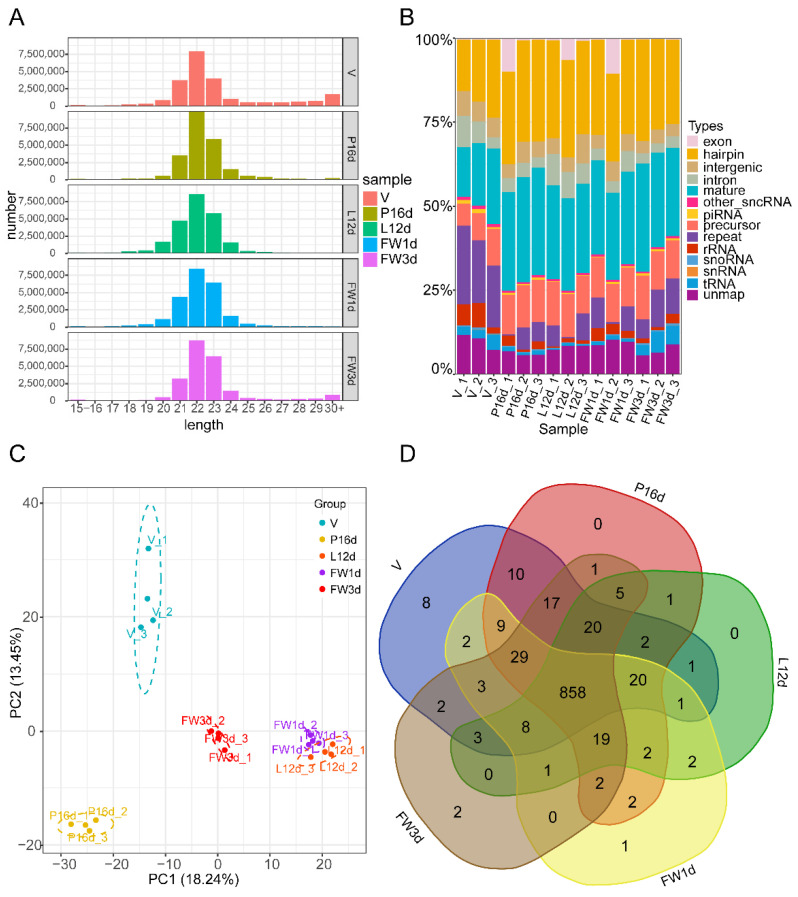
Overview of the small RNA sequencing data: (**A**) Distribution of total identified small RNAs’ lengths. (**B**) Classification of all detected small RNAs. (**C**) Principal component analysis of the total identified miRNAs. (**D**) Venn diagram illustrating the overlap of miRNAs across five different stages.

Following quality control of the sequencing data, each tissue sample obtained an average of over 21 million clean reads (ranging from 20.48 M to 22.95 M). Over 88% of clean reads from each sample were perfectly mapped to the murine genome (GRCm38.p6) (Supplementary Table S2). We examined the read length distribution to evaluate sequencing quality. The majority of small RNAs were clustered between 21 and 23 nt, with a peak distribution of 22 nt (Figure 1A), which is consistent with the sequence length distribution of small RNAs in animal samples. Among the identified small RNAs, the number and proportion of miRNAs—including the mature and hairpin types—in the V, P16d, L12d, FW1d, and FW3d libraries were 5,368,969 (37.66%), 6,485,716 (60.06%), 6,219,803 (55.94%), 6,229,222 (54.61%), and 6,075,677 (56.26%), respectively (Figure 1B). These numbers reflect the inputs to miRNA library preparation. A total of 1031 miRNAs with a TPM ≥ 1 in at least one sample among 15 samples of the 5 different stages were detected, with 852 classified as known miRNAs and 179 identified as novel miRNAs (Supplementary Table S3). Principal component analysis revealed that biological replicates in the five stages were well correlated (Figure 1C). It is worth noting that the murine mammary gland has a similar small RNA expression pattern across the L12d and FW1d stages, which is due to the samples of FW1d having been collected at 24 h of forced weaning, which was performed right after L12d. Although L12d and FW1d samples have an overlap in small RNA expression patterns, subtle small RNA profile differences associated with involution were detected. At the V, P16d, L12d, FW1d, and FW3d stages, 993, 997, 943, 959, and 970 miRNAs were identified, respectively. In these 5 stages, 858 (83.22%) miRNAs were co-expressed (Figure 1D).

### 3.2. Validation of miRNAs Sequence Data with qPCR

**Figure 2:**
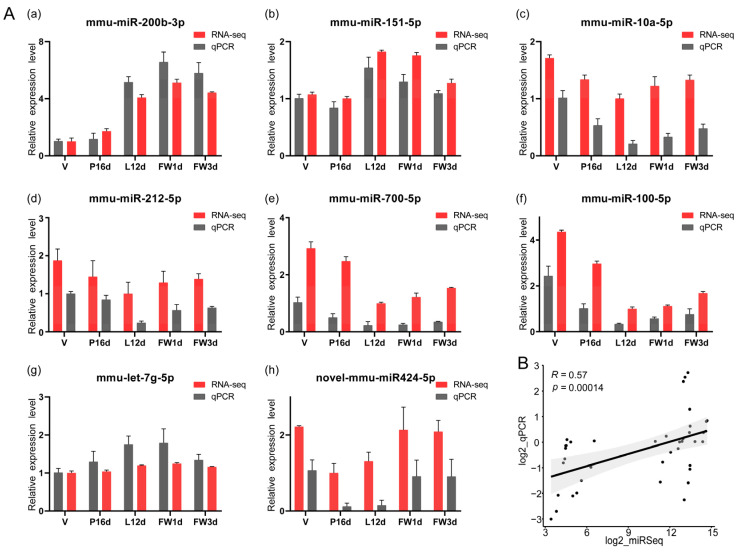
Validation of the expression of miRNAs using qPCR: (**A**) (**a**–**h**). The relative expression levels of eight selected miRNAs measured by qPCR in five stages. The expression levels were normalized against the expression level of the internal control U6 using the comparative cycle threshold (2^−ΔΔCt^) method. Meanwhile, for each miRNA, the stage of lowest sequencing abundance in the five different stages was used as a reference sample for comparisons. Data are presented as the mean ± standard error of the mean (SEM) for three replicates. (**B**) Scatterplot between the qPCR and RNA-Seq results of the eight selected miRNAs. The y-axis corresponds to the log2 of the qPCR ratios, while the x-axis shows the log2 of the RNA-Seq ratios, for the eight selected miRNAs.

To validate the sequencing data, we used stem–loop qPCR to examine the expression levels of eight randomly selected miRNAs (Figure 2A). The findings revealed that the expression levels of these eight miRNAs in the five stages measured by the qPCR were positively correlated with the small RNA sequencing data (Spearman’s R = 0.57, *p* = 0.00014, Figure 2B).

### 3.3. Dynamic Expression Patterns of miRNAs during Mammary Development

**Figure 3:**
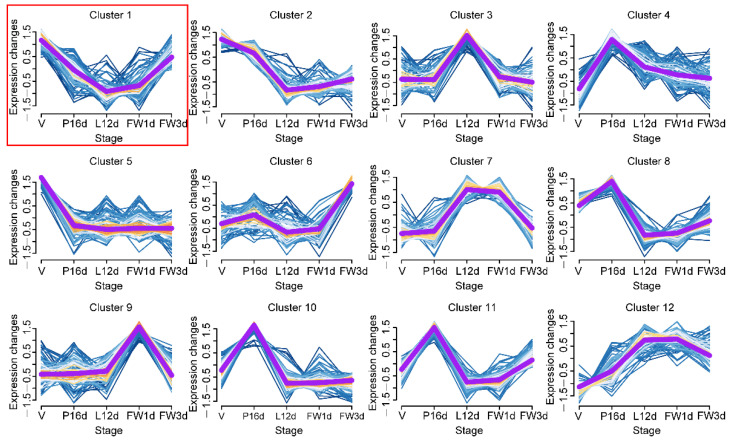
Expression dynamics of 12 miRNA clusters during murine mammary gland development. The purple line represents the miRNAs’ expression trend in each cluster.

MiRNAs with comparable expression patterns throughout murine mammary gland development were clustered using the soft partitioning clustering method of the Mfuzz package [24] to determine the expression patterns of miRNAs in the mammary glands over the five tested developmental stages. Twelve miRNA clusters with different temporal expression profiles were generated (Figure 3 and Supplementary Table S3). We selected cluster 1 with 76 miRNAs for subsequent analysis because miRNAs typically suppress target gene expression, and the expression profile of cluster 1 miRNAs was negatively correlated with that of milk protein genes (Figure 3 and Figure S1B).

### 3.4. Target Gene Predictions for Twelve Clusters miRNAs

A previous work has shown that miRNAs might downregulate target genes’ expression by interacting with the 2–8 bp complementary sequences within the 3′ UTR of the target genes’ transcripts [33]. To enhance prediction accuracy, we utilized three software programs (TargetScan, miRanda, and RNAhybrid) to predict the target genes of 12 miRNA clusters. The predicted target genes of the 12 clusters are shown in Supplementary Table S4. Cluster 1 miRNAs had a total of 9148 predicted target genes (Supplementary Table S4). It is worth noting that *CSN2*—a well-known milk protein gene—was included in the target genes of cluster 1. *CSN2* was predicted to be regulated by the novel-mmu-miR424-5p, which was also found to regulate the largest number of target genes in cluster 1.

### 3.5. Functional Annotation of Cluster 1 miRNAs’ Target Genes

**Figure 4:**
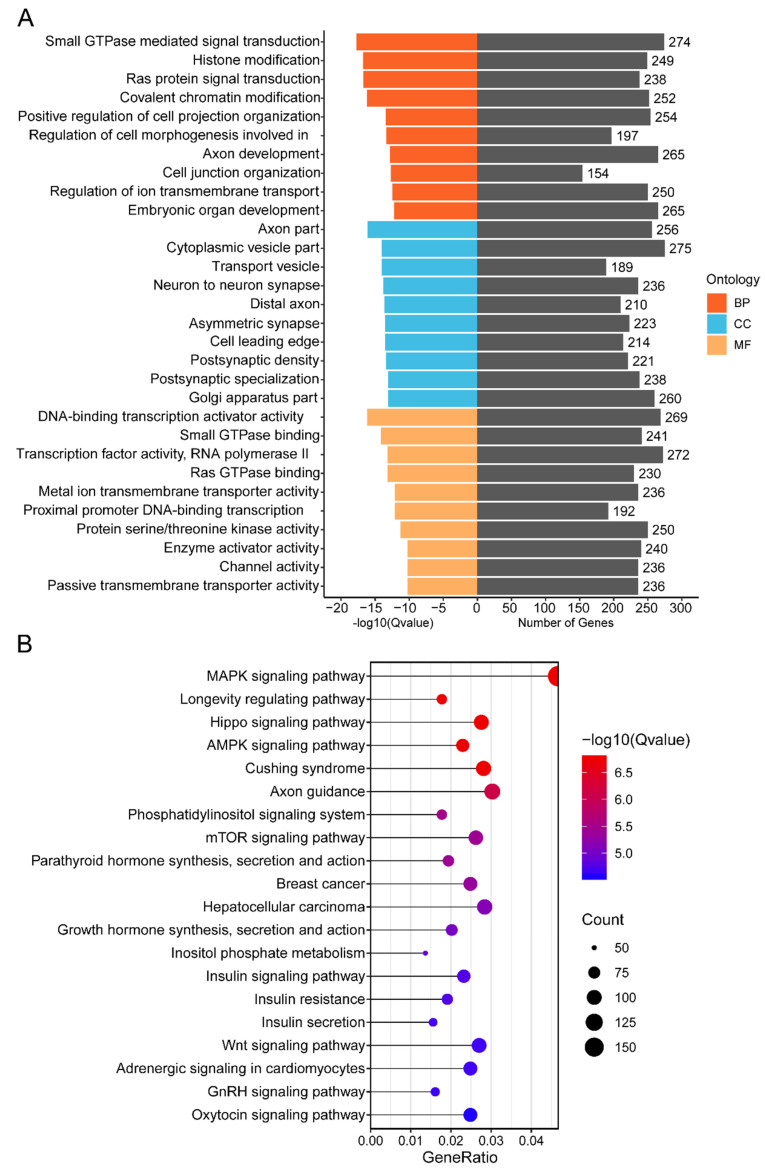
GO and KEGG enrichment analysis of cluster 1 miRNAs’ target genes: (**A**) GO term enrichment analysis of cluster 1 miRNAs’ target genes, including biological process (BP), cellular component (CC), and molecular function (MF). (**B**) KEGG pathway analysis of cluster 1 miRNAs’ target genes. The size of the circle represents the number of genes enriched in the pathway, and the color scale from blue to red (more significant enrichment) represents the enriched significance.

We preformed GO enrichment and KEGG pathway analysis to investigate the functions of cluster 1 miRNAs’ target genes. GO enrichment analysis showed that the target genes were mainly found to be involved in biological processes such as small GTPase-mediated signal transduction (GO:0007264), histone modification (GO:0016570), covalent chromatin modification (GO:0016569), regulation of cell morphogenesis involved in differentiation (GO:0010769), and Ras protein signal transduction (GO:0007265) (Figure 4A; Supplementary Table S5). The KEGG pathway analysis revealed that 324 signaling pathways were enriched (Supplementary Table S6). Among the top 20 signaling pathways, the MAPK signaling pathway (mmu04010), mTOR signaling pathway (mmu04150), parathyroid hormone synthesis, secretion, and action (mmu04928), Hippo signaling pathway (mmu04390), Wnt signaling pathway (mmu04310), and oxytocin signaling pathway (mmu04921) were significantly related to murine mammary gland development (Figure 4B).

### 3.6. Novel-mmu-miR424-5p Expression in Distinct Development Stages Assessed by FISH

**Figure 5:**
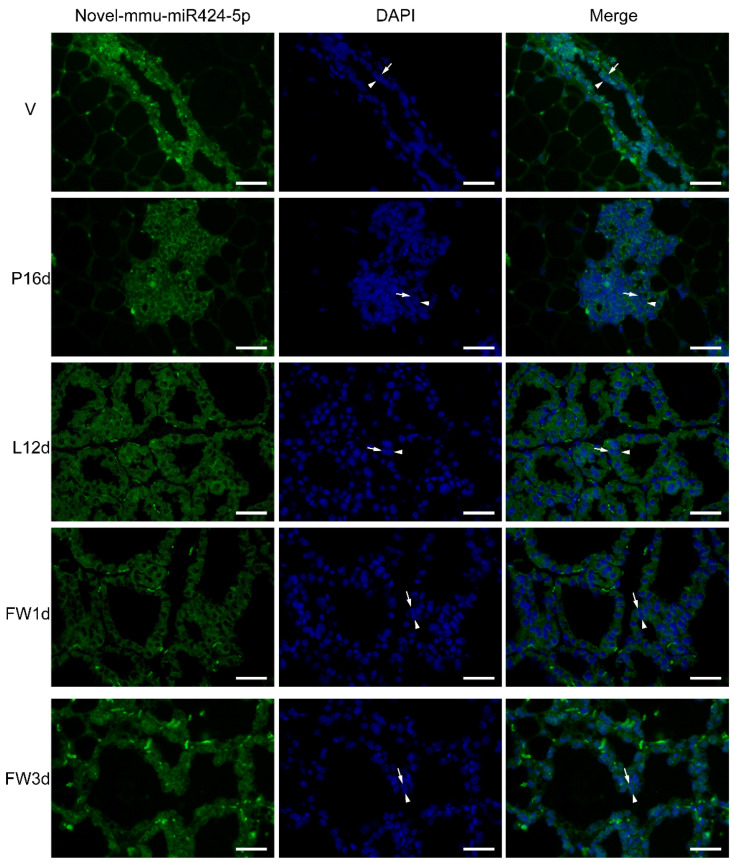
FISH analysis of novel-mmu-miR424-5p at distinct stages of mammary gland development. In the luminal and basal epithelial cell layers, the novel-mmu-miR424-5p was abundantly expressed. The luminal cell is shown by the arrow without a tail, whereas the basal cell is indicated by the arrow with a tail. Scale bars = 20 µm.

FISH analysis was used to identify the location of novel-mmu-miR424-5p in five different stages of mammary gland development. Interestingly, novel-mmu-miR424-5p was found to be significantly expressed in epithelial cells compared to stromal cells. The stromal tissue background signal suggested nonspecific binding in the stromal cells, although the expression of weak miRNAs could not be ruled out. During the virgin stage, the strongest background staining of stromal tissue was observed. Overall, there was a good agreement in the expression patterns between the FISH and the sequencing findings, and novel-mmu-miR424-5p expression was relatively low in the P16d and L12d stages. Interestingly, novel-mmu-miR424-5p was shown to be highly expressed in the luminal cell and basal epithelial cell layers throughout the five stages of development (Figure 5).

### 3.7. Novel-mmu-miR424-5p Directly Targets the 3′UTR of CSN2

**Figure 6:**
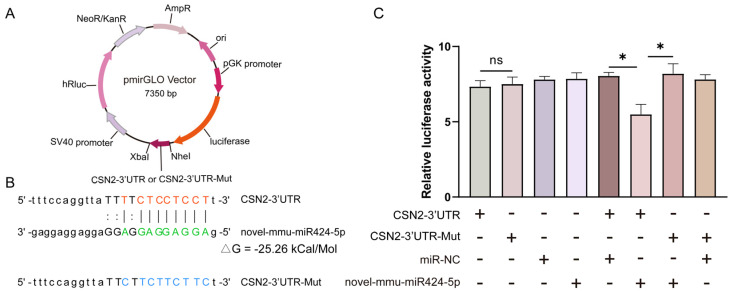
Novel-mmu-miR424-5p targets the 3′ UTR of *CSN2*: (**A**) A map of pmirGLO vectors carrying the predicted WT or Mut target sequences of novel-mmu-miR424-5p in the 3′ UTR of *CSN2*. (**B**) The designed luciferase reporter sequences—WT: the WT *CSN2*-3′UTR sequence includes a novel-mmu-miR424-5p binding site; Mut: the sequence of *CSN2*-3′UTR with mutation in the novel-mmu-miR424-5p binding site. (**C**) Luciferase activity in 293T cells co-transfected with WT or Mut *CSN2*-3′UTR luciferase reporter and novel-mmu-miR424-5p mimics or negative control mimics (miR-NC). +: the vector or mimics was transfected in cells; −: the vector or mimics was not transfected in cells. The relative amounts of firefly luminescence normalized to *Renilla luminescence* are plotted. Data are shown as mean ± SEM values (*n* = 3, * *p* < 0.05, Student’s *t*-test).

According to the bioinformatics predictions of target genes, the novel-mmu-miR424-5p can target an important milk protein gene—*CSN2* (Supplementary Table S4). To verify this prediction, the pmirGLO vector was designed to include the predicted 3′ UTR target sequences for *CSN2* and the 3′ UTR-Mut sequences for *CSN2* (Figure 6A,B). Using the dual-luciferase reporter assays, the targeting relationship between novel-mmu-miR424-5p and the 3′UTR of *CSN2* was assessed. The luciferase levels in 293T cells transfected with *CSN2*-3′UTR and *CSN2*-3′UTR-Mut luciferase reporter were comparable (*p* = 0.80) (Figure 6C). The luciferase level in 293T cells co-transfected with *CSN2*-3′UTR and novel-mmu-miR424-5p mimics was significantly lower than that in 293T cells co-transfected with *CSN2*-3′UTR and negative control mimics (miR-NC) (*p* = 0.02), or in 293T cells co-transfected with *CSN2*-3′UTR-Mut and novel-mmu-miR424-5p mimics (*p* = 0.04). This confirms that one of the likely targets of novel-mmu-miR424-5p is the 3′UTR of *CSN2*, which suggests that novel-mmu-miR424-5p may play a critical role in the process of milk production during mammary development.

## 4. Discussion

miRNAs, as key gene expression regulators, have been shown to play crucial roles in regulating mammary gland development and lactation [34,35,36]. However, the overall dynamics of miRNAs in the process of mammary gland differentiation have not been clearly elucidated. This is the first study to comprehensively report miRNAs’ dynamics throughout five different stages of murine mammary gland development. A total of 852 known murine miRNAs and 179 novel murine miRNAs were identified in this study, expanding the repertoire of murine mammary-gland-expressed miRNAs, and the identified developmental-stage-specific miRNAs provide a valuable resource for understanding the role of miRNAs during murine development.

We found that 858 miRNAs, accounting for 83.22% of identified total miRNAs, were co-expressed in the five stages, indicating that most miRNAs were expressed among the five stages. Notably, eight, one, and two miRNAs were specifically identified at the V, FW1d, and FW3d stages, respectively. This indicates that the 11 corresponding molecules may play vital roles in specific developmental stages. The blood vessels in the mammary glands undergo a process of formation and expansion during pregnancy and lactation, which is an important event during mammary gland development [37]. miR-208a-3p inhibits the formation of vascular tissues by targeting the mRNA and its target genes [38]. Therefore, miR-208a-3p should be maintained at a low expression level during pregnancy and lactation in order to ensure normal angiogenesis in the mammary glands. These data are in agreement with in the findings of this study, as miR-208a-3p was classified as a cluster 5 miRNA, and *FGF14* is one of its predicted target genes, which was shown to be involved in regulating angiogenesis [39]. Therefore, we have more reason to believe that miR-208a-3p may play a key role in mammary gland development.

In the study by Guillou et al. [40], there were 824 known murine miRNAs identified in the L12d mammary glands of FVB/N mice using RNA-Seq, and 575 of them were among the 783 known miRNAs identified at the L12d stage in the present study. Interestingly, both Guillou’s study and our study found six miRNAs (miR-126-5p, miR-16-5p, miR-141-3p, miR-200a-3p, miR-200b-3p, and miR-200c-3p) to be highly expressed at the L12d stage. These six miRNAs participate in regulating the tight junction pathway involved in cellular structure in mammary gland biology [40]. For the previously identified top 10 miRNAs enriched in the terminal end-buds or mature ducts of the pubertal murine mammary glands [41], 9 of them (mmu-miR-31, mmu-miR-17, mmu-miR-18a, mmu-miR-362-5p, mmu-miR-19a, mmu-miR-184, mmu-let-7g, mmu-miR-346, and mmu-miR-328), excluding only mmu-miR-1894-5p, were identified in this study, and were found to be expressed in all five stages. miR-18a-5p has been shown to regulate lipid metabolism by targeting the expression of sterol regulatory element-binding transcription protein 1 (*SREBP1*) [42], which could form a complex with Snail to modulate epithelial–mesenchymal transition (EMT) in pubertal mammary glands [42,43]. In this study, miR-18a-5p was enriched in cluster 11, and was predicted to regulate 55 target genes. Although *SREBP1* was not predicted as a target gene of miR-18a-5p, *SLC22A5,* as one of the predicted target genes, also plays roles in lipid metabolism [44], and *IKBKE*, as another target gene, directly phosphorylated Snail to EMT in a recent study [45]. Therefore, we speculate that miR-18a-5p may participate in the regulation of lipid metabolism and EMT in mammary glands by regulating several target genes. Furthermore, miR-31 has been shown to regulate the self-renewal/proliferation of basal cells and inhibit their differentiation into luminal/alveolar cells by activating the Wnt/β-catenin signaling pathway in mammary glands [46]. In this study, the miR-31 was enriched in cluster 6, and *DAPK2* was predicted as a target gene of miR-31. A previous study has shown that *DAPK2* regulates apoptosis [47]. Therefore, we surmise that miR-31 may be involved in regulating mammary gland development.

All of the miRNAs identified in this study can be classified into 12 distinct clusters, indicating that the identified miRNAs may play a variety of roles in regulating mammary gland development. Notably, only the expression pattern of cluster 1 miRNAs was opposite to that of milk protein genes, implying that some miRNAs in cluster 1 may be involved in regulating the expression of milk protein genes. Among the miRNAs found in this study, two previously reported miRNAs—miR-15b and miR-223—are notable. miR-15b was enriched in cluster 11, while miR-223 was enriched in cluster 6, both of which were differentially expressed in the five stages. miR-15b and miR-223 regulate lipid synthesis and milk production, and ductal morphogenesis, respectively [16,17]. The expression trends of these two miRNAs in the five stages were consistent with their function in mammary glands. Furthermore, some previously identified differentially expressed miRNAs during pregnancy and lactation—including miR-10b, miR-152, miR-145, and miR-21—were found to be enriched in cluster 1 [48]. A previous study showed that miR-145 regulates cell proliferation and differentiation [49], while luciferase reporter assays confirmed that rhotekin (*RTKN*) is a direct target of miR-145 [50]. *RTKN* plays roles in regulating cell growth [51]. In addition, upregulation of *RTKN* expression activates the Wnt/β-catenin pathway [50], which regulates the developmental fate of mammary epithelium cells [52]. In this study, the expression of miR-145 in five stages decreased gradually throughout the V, P16d, and L12d stages, and then increased gradually in the FW1d and FW3d stages. Meanwhile, *RTKN* is predicted as the one of target genes of miR-145. We believe that miR-145 may target *RTKN*, acting on the Wnt/β-catenin signaling pathway to regulate cell fate in mammary gland development. However, this conjecture needs to be verified by further research.

Among the top 10 biological processes identified by GO enrichment analysis for cluster 1 miRNAs’ predicted target genes, most of them were related to mammary gland development and lactation, including small GTPase-mediated signal transduction, Ras protein signal transduction, regulation of cell morphogenesis involved in differentiation, histone modification, and covalent chromatin modification [53,54,55]. Using KEGG pathway analysis, several cluster 1 miRNAs’ target genes were integrated into the Wnt signaling pathway, mTOR signaling pathway, MAPK signaling pathway, and Hippo signaling pathway. The Wnt signaling pathway is reported to play an essential role in initiating mammary gland morphogenesis and accelerating mammary gland development [56]. Meanwhile, this pathway has been found to contribute to mammary gland growth, differentiation, lactation, and involution by regulating cell processes [57]. Furthermore, the MAPK signaling plays an important role in regulating the processes of MECs’ proliferation, differentiation, apoptosis, and migration [58], and the Hippo pathway is indispensable in the virgin mammary glands, but particularly needed during pregnancy, when MECs undergo rapid growth and differentiation [59]. mTOR has been shown to play a role in regulating milk protein synthesis [60]. Cluster 1 miRNAs’ target genes were also shown to be enriched in several hormone-related signaling pathways, including growth hormone, insulin, oxytocin, and parathyroid hormone, all of which are implicated in the regulation of mammary gland proliferation, differentiation, and lactation [4,61,62]. These findings suggest that cluster 1 miRNAs may play an important role in the regulation of murine mammary gland development and lactation.

The expression of milk protein genes is regulated by tissue- and stage-specific genes in the mammary glands [63]. β-casein is a vital source of essential amino acids and calcium for suckling pups [64], and forms micelle structures with other casein subtypes. In particular, αs1-casein and k-casein contribute to efficient secretion of β-casein [65,66]. β-casein accounts for ~20% of total milk proteins; it is a common milk protein in mammalian species, and is an ideal marker for mammary differentiation [67,68]. *CSN2* is expressed in mid-pregnancy, and then increases in expression, and is confined to the lactating mammary gland epithelial cells [69]. Previous studies have found that transcription factors, growth factors, and hormones all influence *CSN2* expression [70,71,72]. Meanwhile, research has shown that miR-139 regulates *CSN2* synthesis in bovine mammary epithelial cells by targeting the mRNAs of GHR and IGF1R [73], while miR-101a inhibits *CSN2* expression by regulating cyclooxygenase-2 expression [74]. In this study, we found that one of the likely targets of novel-mmu-miR424-5p is the 3′UTR of *CSN2*. This indicates that novel-mmu-miR424-5p plays an important role in murine mammary gland development and lactation, and it may be functionally defined as a negative regulator of mammary differentiation and milk protein expression.

However, we only confirmed that the 3′UTR of *CSN2* is a target of novel-mmu-miR424-5p. Future research is needed in order to investigate whether the novel-mmu-miR424-5p can suppress *CSN2* expression in murine mammary gland epithelial cells in vitro or in vivo. Furthermore, the miRNAs in the other 11 clusters—particularly clusters 7 and 12—may be important in regulating mammary gland development and milk protein expression, because the expression patterns of these cluster miRNAs were comparable to those of milk protein genes, and the miRNAs of these clusters may promote the expression of milk protein genes by targeting their inhibitors. Therefore, miRNAs in clusters 7 and 12 should be further investigated in future research.

## 5. Conclusions

This study revealed the dynamic characteristics of miRNAs’ expression, and identified 852 known miRNAs and 179 novel miRNAs in murine mammary glands throughout five important developmental stages. The target genes of a cluster of identified cluster 1 miRNAs, with an expression profile negatively correlated with that of milk protein genes, were enriched in singling pathways related to mammary gland development and lactation. In cluster 1 miRNAs, a novel miRNA—the novel-mmu-miR424-5p—was identified as a potential negative regulator of expression of the key milk protein gene *CSN2*. Therefore, our findings provide a basis for an in-depth understanding of the murine mammary gland’s developmental regulation, which will be helpful for further research on human breast disease and animal husbandry.

## Data Availability

The datasets used and analyzed in this study can be found in online repositories. The raw reads produced in this study were deposited in the NCBI Sequence Read Archive (SRA), accession number PRJNA767953.

## Supplementary Materials

The following are available online at https://www.mdpi.com/article/10.3390/ani12060727/s1: Supplementary Figure S1: The histological characteristics and expression profiles of milk protein genes in the murine mammary gland at five different stages; Supplementary Table S1: Primers used in the present study; Supplementary Table S2: The proportion of reads mapped to the reference genome; Supplementary Table S3: TPM of miRNAs from murine mammary glands at five distinct stages; Supplementary Table S4: Target gene predictions for 12 miRNA clusters; Supplementary Table S5: GO enrichment analysis of cluster 1 miRNAs’ target genes; Supplementary Table S6: KEGG pathway analysis of cluster 1 miRNAs’ target genes.
