# Supplementary material for: Dynamic miRNA Landscape Links Mammary Gland Development to the Regulation of Milk Protein Expression in Mice
Source: Animals (Basel). 2022 Mar 14;12(6):727. doi: 10.3390/ani12060727 (PMC8944794; doi:10.3390/ani12060727)
Supplement: Supplementary file 1 [file animals-12-00727-s001.zip › Supplementary Table S1.pdf]

Supplementary Table S1 Primers used in the present study

Name	Sequence (5′- 3′)
Primers designed to quantify mRNA	
α-casein-F	TGCAGCATCTGAGGAACAAG
α-casein-R	TCTGAAGCTGTTCCAGGGTG
β-casein-F	GGTGAATCTCATGGGACAGC
β-casein-R	CACAGGGGGTTGAGCAATAG
Wap-F	GGCTTCTGCCCTTGGAATCT
Wap-R	TGAGACTGCATACCTGGCAC
β-actin -F	TGAGCTGCGTTTTACACCCT
β-actin -R	GCCTTCACCGTTCCAGTTTTT
Primers designed to quantify miRNA	
mmu-miR-212-5p-RT	GTCGTATCCAGTGCAGGGTCCGAGGTATTCGCACTGGATACGACAGTAAG
mmu-miR-212-5p-F	ATGGGACCTTGGCTCTAGACT
mmu-miR-212-5p-R	CAGTGCAGGGTCCGAGGTAT
mmu-miR-200b-3p-RT	GTCGTATCCAGTGCAGGGTCCGAGGTATTCGCACTGGATACGACTCATCA
mmu-miR-200b-3p-F	ACGGGCTAATACTGCCTGGTAA
mmu-miR-200b-3p-R	CAGTGCAGGGTCCGAGGTAT
mmu-let-7g-5p-RT	GTCGTATCCAGTGCAGGGTCCGAGGTATTCGCACTGGATACGACAACTGT
mmu-let-7g-5p-F	ACGGGCTGAGGTAGTAGTTTGT
mmu-let-7g-5p-R	CAGTGCAGGGTCCGAGGTAT
mmu-miR-151-5p-RT	GTCGTATCCAGTGCAGGGTCCGAGGTATTCGCACTGGATACGACACTAGA
mmu-miR-151-5p-F	CAAATTCGAGGAGCTCACAGT
mmu-miR-151-5p-R	CAGTGCAGGGTCCGAGGTAT
mmu-miR-10a-5p-RT	GTCGTATCCAGTGCAGGGTCCGAGGTATTCGCACTGGATACGACCACAAA
mmu-miR-10a-5p-F	GGGTACCCTGTAGATCCGAAT
mmu-miR-10a-5p-R	CAGTGCAGGGTCCGAGGTAT
mmu-miR-100-5p-RT	GTCGTATCCAGTGCAGGGTCCGAGGTATTCGCACTGGATACGACCACAAG

mmu-miR-100-5p-F	GGAACCCGTAGATCCGAACT
mmu-miR-100-5p-R	CAGTGCAGGGTCCGAGGTAT
mmu-miR-700-5p-RT	GTCGTATCCAGTGCAGGGTCCGAGGTATTCGCACTGGATACGACGCAAGC
mmu-miR-700-5p-F	ACCCTAAGGCTCCTTCCTGTG
mmu-miR-700-5p-R	CAGTGCAGGGTCCGAGGTAT
novel-mmu-miR424-5p-RT	GTCGTATCCAGTGCAGGGTCCGAGGTATTCGCACTGGATACGACCTCCTC
novel-mmu-miR424-5p-F	GCAAATGAGGAGGAGGAGGA
novel-mmu-miR424-5p-R	CAGTGCAGGGTCCGAGGTAT
mmu-U6-RT	GGGCCATGCTAATCTTCTCTG
mmu-U6-F	TCGCTTCGGCAGCACATA
mmu-U6-R	GGGCCATGCTAATCTTCTCTG
