# Supplementary figures and images for: Dynamic miRNA Landscape Links Mammary Gland Development to the Regulation of Milk Protein Expression in Mice
Source: Animals (Basel). 2022 Mar 14;12(6):727. doi: 10.3390/ani12060727 (PMC8944794; doi:10.3390/ani12060727)

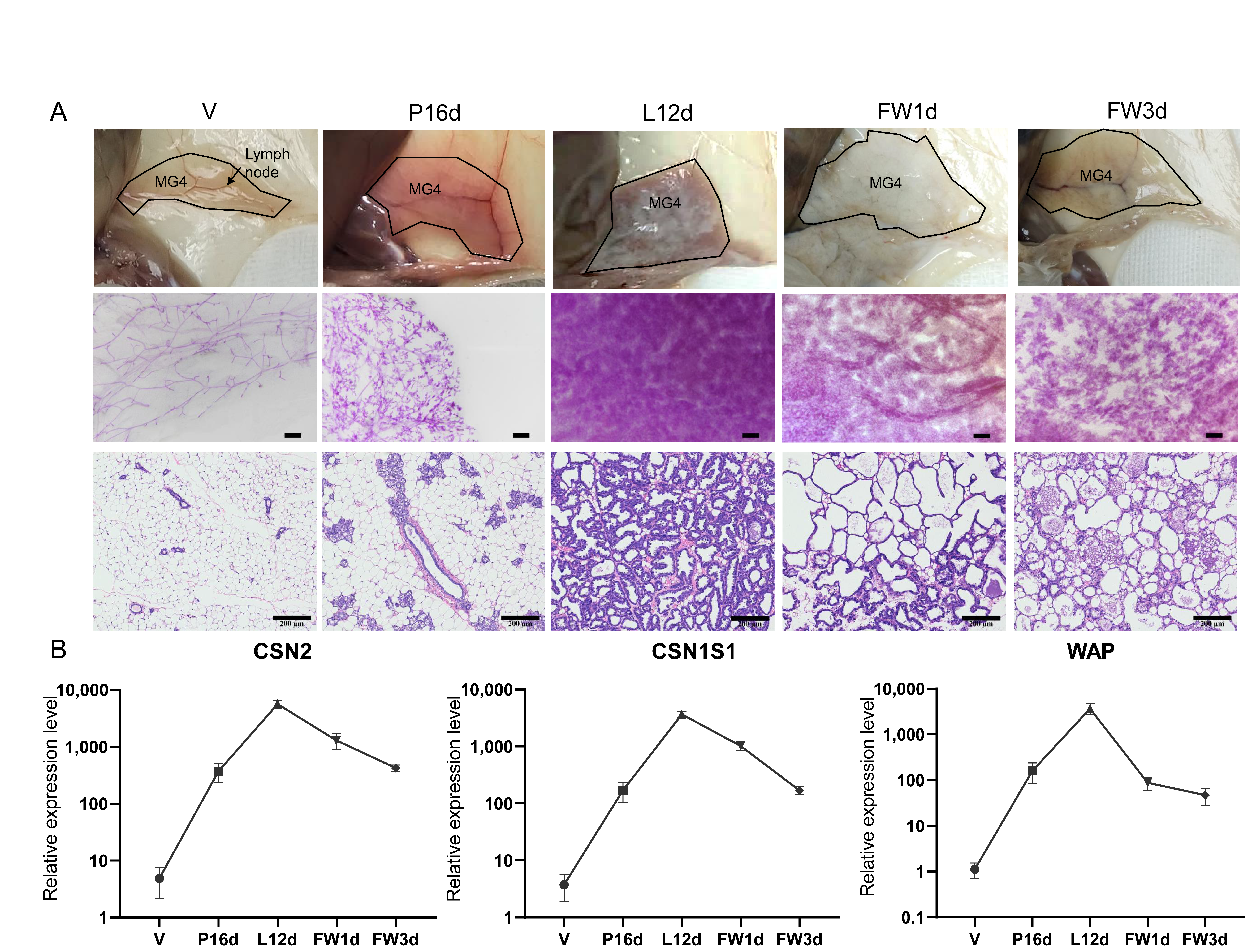

Supplement: Supplementary file 1 [file animals-12-00727-s001.zip › Supplementary Figure S1.tif]
